# Dataset on the kinetics of the inhibition of PTP1B by the flavonoids and pheophytin A from *Allophylus cominia*

**DOI:** 10.1016/j.dib.2018.01.057

**Published:** 2018-02-03

**Authors:** D.G. Semaan, J.O. Igoli, L. Young, E. Marrero, A.I. Gray, E.G. Rowan

**Affiliations:** a Institute of Pharmacy and Biomedical Sciences, University of Strathclyde, 161 Cathedral Street, Glasgow G4 0RE, United Kingdom; b National Centre for Animal and Plant Health (Centro Nacional de Sanidad Agropecuaria), San José de las Lajas, Mayabeque, Cuba; c Department of Chemistry, University of Agriculture, PMB 2373 Makurdi, Nigeria

**Keywords:** Flavonoids, Pheophytin, Inhibition, Kinetics, PTP1B enzyme

## Abstract

The data presented in this article are related to the research article under the title “in vitro anti-diabetic activity of flavonoids and pheophytins from *Allophylus cominia* Sw. on PTP1B, DPPIV, alpha-glucosidase and alpha-amylase enzymes” (Semaan et al., 2017) [3]. This article defines the kinetics of inhibition of flavonoids and pheophytin A extracts from *A. cominia* which showed an inhibition of the PTP1B enzyme activity. The main reason to make these results public is to confirm that this study was followed up and no more experiments are needed, also to confirm that these compounds can be reported as PTP1B inhibitors.

**Specifications Table**

**Table:** 

Subject area	*Enzymology*
More specific subject area	*Enzyme kinetic studies*
Type of data	*Figures and tables*
How data was acquired	*in vitro assays*
Data format	*Analyzed*
Experimental factors	*All enzymes, substrates and inhibitors were prepared freshly before each experiment*
Experimental features	*Each experiment were repeated three times*
Data source location	*United kingdom, Glasgow, University of Strathclyde*
Data accessibility	*The data are accessible only within this article*

**Value of the data**

• The data presented in this article is original and have not been published before.
• The data presents the importance of the samples extracted from the Cuban plant on the inhibition of PTP1B.
• The data can be used by other researches to follow on with more studies regarding the mechanism of action of these samples.

## 1 Data

**Fig. 1:**
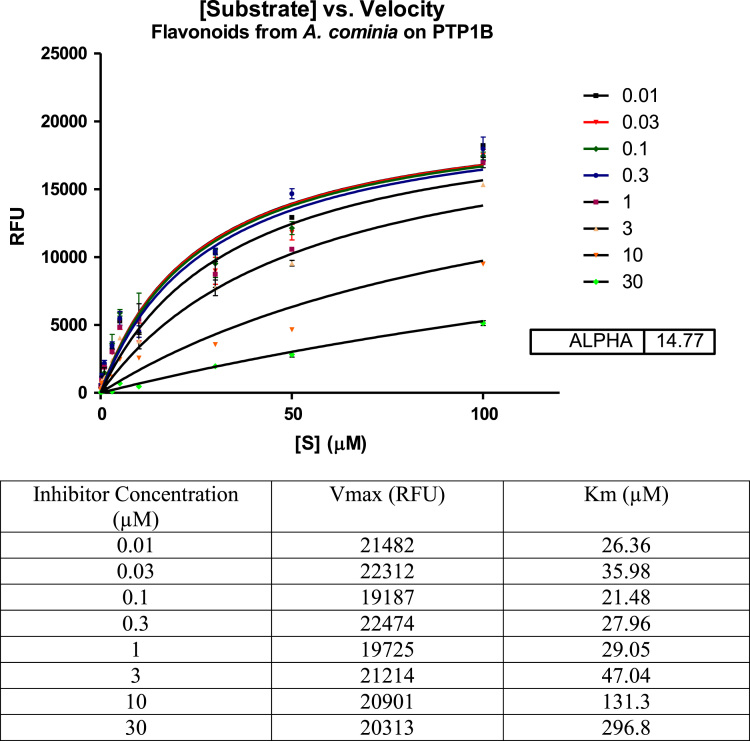
Michaelis-Menten plot of the inhibitory effect of the flavonoid fractions of *A. cominia* on PTP1B-catalysis hydrolysis of the enzyme. Data are expressed as mean RFU (relative fluorescence unit) for *n* = 3 replicates of each substrate concentration (0.01–100 µM). The table below the graph represents *K*_m_ (µM) and *V*_max_ (RFU) with each inhibitor concentration.

**Fig. 2:**
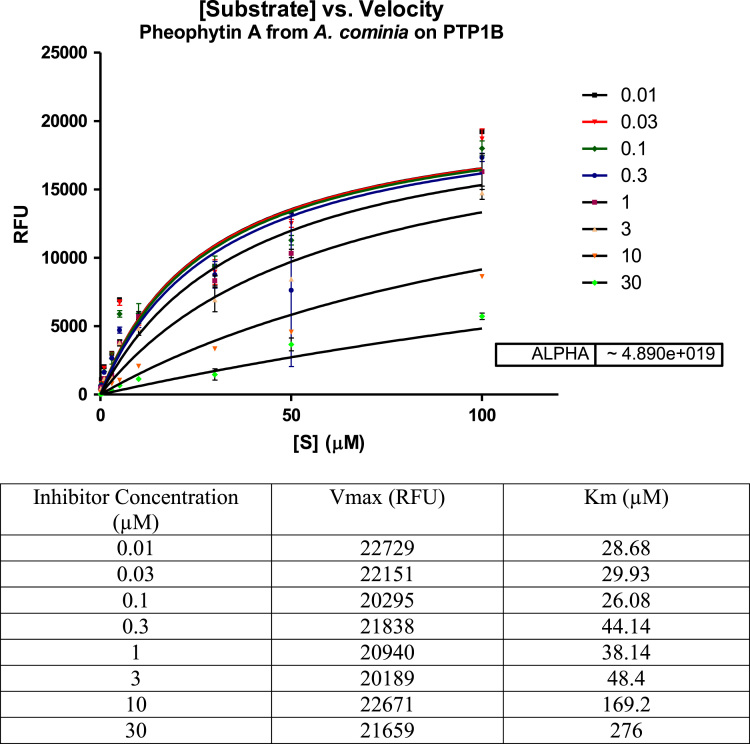
Michaelis-Menten plot of the inhibitory effect of the pheophytin A fraction of *A. cominia* on PTP1B-catalysis hydrolysis of the enzyme. Data are expressed as mean RFU (relative fluorescence unit) for *n* = 3 replicates of each substrate concentration (0.01–100 µM). The table below the graph represents *K*_m_ (µM) and *V*_max_ (RFU) with each inhibitor concentration.

Various concentrations of the flavonoids and pheophytin A samples were incubated with PTP1B enzyme and increasing concentrations of the substrate. The results were graphed using a Michaelis-Menten plot. *V*_max_ and *K*_m_ were calculated (Fig. 1, Fig. 2). As the flavonoids and pheophytin A concentration increased, so did the *K*_m_. The *V*_max_ was unchanged with increased concentrations of inhibitors.

The shape of the curves in the Michaelis-Menten plot was hyperbolic. Alpha was > 1 for both samples. All these factors were indicative of a competitive inhibition of the flavonoid and pheophytin A samples.

The mechanism of action of the TFMS inhibitor used in the assay also confirmed its competitive inhibition. As the TFMS concentration increased, so did the *K*_m_. The *V*_max_ was unchanged with increased concentrations of inhibitor.

**Fig. 3:**
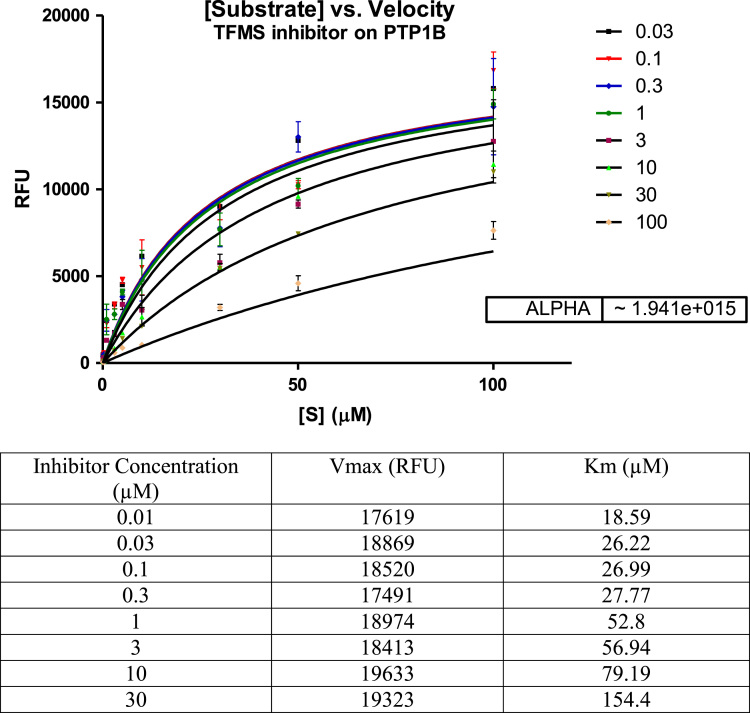
Michaelis-Menten plot of the inhibitory effect of the TFMS on PTP1B-catalysis hydrolysis of the enzyme. Data are expressed as mean RFU (relative fluorescence unit) for *n* = 3 replicates of each substrate concentration (0.01–100 µM). The table below the graph represents *K*_m_ and *V*_max_ with each inhibitor concentration.

The shape of the curve in the Michaelis-Menten plot was hyperbolic. Alpha was around 1.941e+015 > 1 (very large) (Fig. 3). The mechanisms of action of flavonoid and pheophytin A samples extracted from *Allophylus cominia* were comparable to that of the TFMS inhibitor of PTP1B enzyme activity [1], [2], [4].

## 2 Experimental design, materials and methods

Various concentrations of *A. cominia* extract (both flavonoids and pheophytins, concentration range 0.01–30 µg/ml) were incubated with PTP1B enzyme (2 nM) at 37 °C for 30 min [3]. Various concentrations of PTP1B substrate (DiFMUP, concentration range 0–40 µM) was added and incubated at 37 °C in an atmosphere containing 5% CO_2_ for another 10 min. The mechanism of inhibition of PTP1B by *A. cominia* extract was compared with the commercial inhibitor P32/98. The same procedure was repeated with various concentrations of the PTP1B inhibitor (TFMS, concentration range 0.0003–3 µM). The umbelliferone was tested using a Wallac Victor^2^ using ex 355 nm/em 460 nm.
